# Evaluation of the Interaction between Long Telomeric DNA and Macrocyclic Hexaoxazole (6OTD) Dimer of a G-quadruplex Ligand

**DOI:** 10.3390/molecules18044328

**Published:** 2013-04-12

**Authors:** Keisuke Iida, Satoki Majima, Takahiro Nakamura, Hiroyuki Seimiya, Kazuo Nagasawa

**Affiliations:** 1 Faculty of Technology, Tokyo University of Agriculture and Technology (TUAT), 2-24-16 Naka-cho, Koganei-shi, Tokyo 185-0031, Japan; 2 Cancer Chemotherapy Center, Japanese Foundation for Cancer Research (JFCR), 3-8-31 Ariake, Koto-ku, Tokyo 135-8550, Japan

**Keywords:** macrocycles, G-quadruplex, telomestatin, telomere, oxazole

## Abstract

Macrocyclic hexaoxazole dimer of L2H2-6OTD-dimer (**3**) was newly synthesized as a telomeric G-quadruplex (G4) ligand, and interaction with long telomeric DNAs telo48, 72, and 96 was evaluated by means of electrophoresis mobility shift assay, CD spectra analysis, and CD melting experiments. The L2H2-6OTD-dimer (**3**) interacted with the long telomeric DNAs by inducing anti-parallel type G4 structure of each unit of 24 bases,* i.e.*, (TTAGGG)_4_ sequences. Dimer **3** stabilizes long telomeric DNAs more efficiently than the corresponding monomer of L2H2-6OTD (**2**). It showed potent inhibitory activity against telomerase, with an IC_50_ value of 7.5 nm.

## 1. Introduction

**Figure 1:**
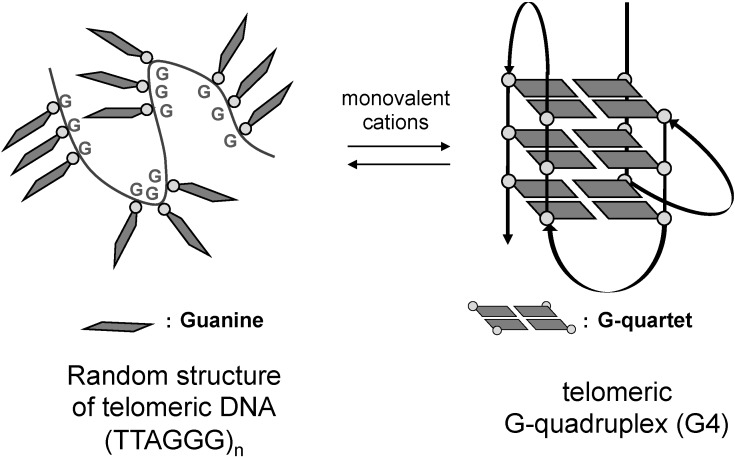
Induction of G-quadruplex formation on telomeric DNA by monovalent cations.

**Figure 2:**
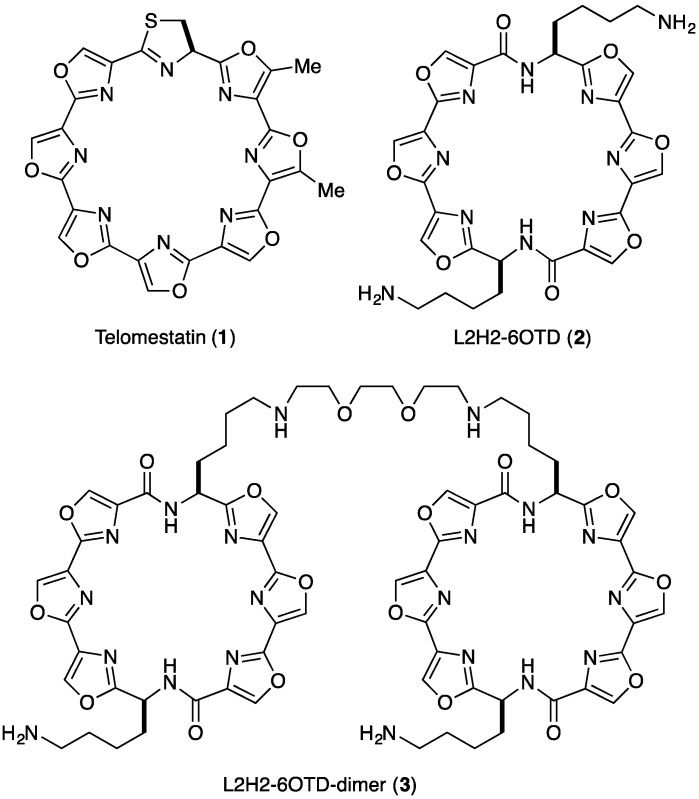
.Structures of telomestatin (**1**), L2H2-6OTD (**2**) and L2H2-6OTD-dimer (**3**).

At the end of chromosomes, there exists a characteristic region called the telomere. The telomere is composed of double-stranded tandem repeats of (TTAGGG/AATCCC)_n_ sequence amounting to 2–30 kb, to which telomere binding proteins are bound [1,2,3,4]. These sequences are shortened at each cell division, ultimately inducing cell senescence. At the extreme end of these duplex repeats there is a single-stranded 3' overhang, termed the 3' G-tail, consisting of ca. 50−250 nucleotides in total. The G-tail sequence is considered to form G-quadruplexes (G4s), one of the higher-order structures of polynucleotides [5]. The G4s are stacked structures of planar G-quartets, consisting of four guanine residues, and these structures are in equilibrium with random structures in the presence of monovalent cations such as Na^+^ and K^+^ (Figure 1) [6,7,8,9]. In most cancer cells, the enzyme telomerase is over-expressed. This enzyme catalyzes elongation of telomeric DNA by adding (TTAGGG) repeats in the 3' terminus direction, leading to immortalization of the cells [10,11]. On the other hand, the telomeric G4 structures in cancer cells inhibit telomerase activity, leading to cellular senescence and apoptosis [12,13,14]. Thus, stabilization of telomeric G4 structure by small molecules,* i.e.*, G4 ligands, is considered to be a promising strategy for cancer chemotherapy, and various G4 ligands, both natural and synthetic, have been explored [15,16]. Among them, telomestatin (Figure 2a), a secondary metabolite isolated from *Streptomyces anulatus*, was found to show extremely potent telomerase inhibitory activity with an IC_50_ value of 5 nm in TRAP assay [17,18,19,20,21,22]. Consequently, various telomestatin-related macrocyclic polyoxazole compounds have been synthesized [23,24,25,26,27,28,29,30]. We have independently developed macrocyclic hexaoxazole-type telomestatin derivatives of 6OTDs as G4 ligands, and some of them showed potent telomerase-inhibitory activity, as well as G4-stabilizing activity (Figure 2a) [31,32,33,34,35,36,37,38,39,40,41]. 

**Figure 3:**
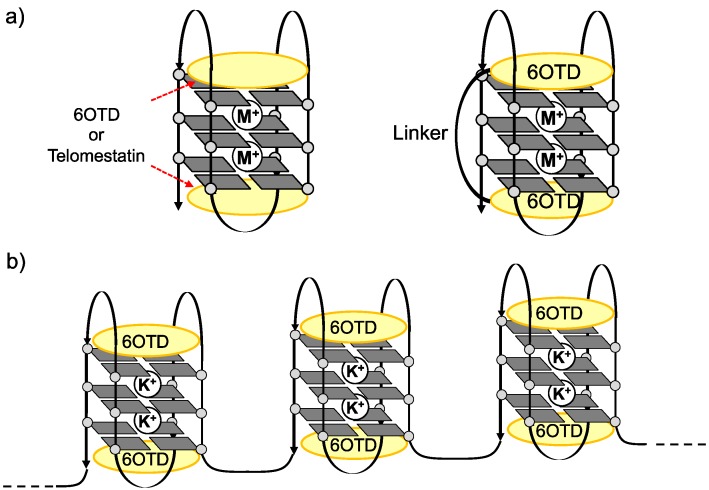
(**a**) Schematic interaction model of telomestatin (**1**), L2H2-6OTD (**2**) and L2H2-6OTD-dimer (**3**)with telo24; (**b**) Schematic interaction model of L2H2-6OTD (**2**) with long telomeric DNA.

For evaluations of interaction and stabilization of telomeric DNA with G4 ligands, telo24 DNA,* i.e.*, four sets of (TTAGGG) repeats consisting of 24 bases in total, has been usually used as a telomeric DNA model, because it is a minimum length able to form one unit of G4 structure [7,8]. L2H2-6OTD (**2**) (Figure 2), a derivative of the 6OTD series with an amine group on the side chain, strongly interacts with telo24 to form anti-parallel-type G4 structure in an end-stacking mode with two molecules of **2** (Figure 2). However, telomeric DNA possesses *ca*. 50−250 bases, so that longer telomeric DNA models are required to mimic the behavior of telomeres in living cells [42,43,44,45,46,47,48,49,50,51,52]. Quite recently, we examined the interaction of L2H2-6OTD (**2**) with long telomeric DNAs telo48, 72, and 96, and these were found to be stabilized in a similar manner to telo24,* i.e.*, each telo24 unit in the long telomeric DNAs interacted with two molecules of L2H2-6OTD (**2**) in an end-stacking mode, and were induced to take anti-parallel-type topology, as shown in Figure 3b [53]. Based on this schematic interacting model of L2H2-6OTD (**2**) with long telomeric DNAs, we expected that the dimer-type of 6OTD might more effectively stabilize the “flexible” long telomeric DNA by interaction of one molecule of 6OTD-dimer with each telo24 unit in a sandwich manner (Figure 3) [36,37,38]. In the present work, we evaluated the interaction mode and efficacy of L2H2-6OTD-dimer (**3**) with long telomeric DNAs by means of electrophoresis mobility shift assay (EMSA), circular dichroism (CD) titration analysis, CD melting experiments and TRAP assay.

## 2. Results and Discussion

**Scheme 1:**
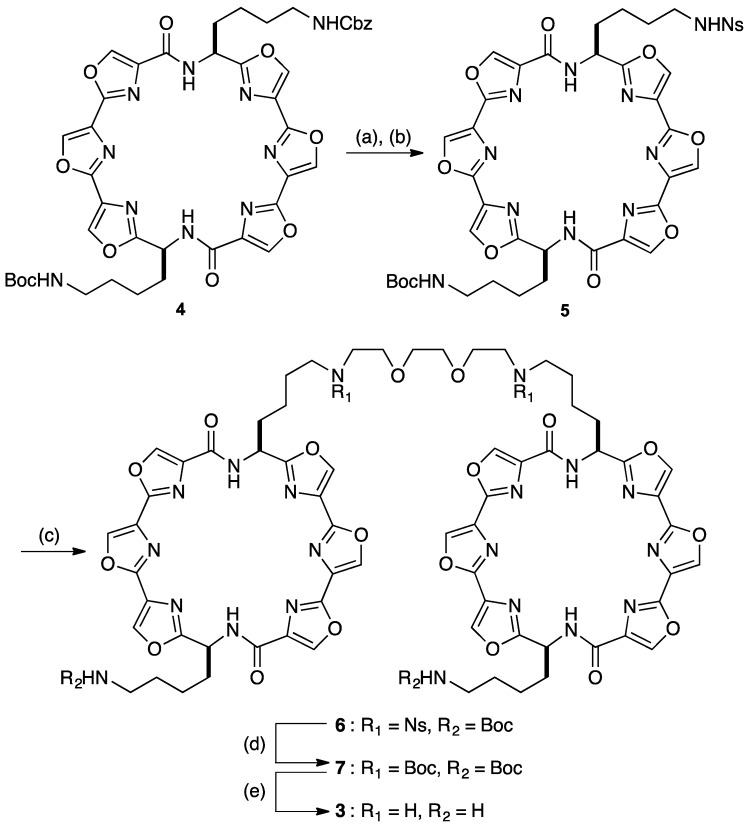
Synthesis of the L2H2-6OTD-dimer (**3**).

We designed the L2H2-6OTD-dimer (**3**) based upon our previous findings, which suggested that it requires about 10–15 Å linker length to interact with one unit of telomeric G4 of telo24 [36]. We chose the triethylene glycol unit as the linker because of its flexibility and hydrophilic nature. Synthesis of L2H2-6OTD dimer (**3**) is depicted in Scheme 1. The Cbz group in diamine **4** [37] was selectively deprotected with hydrogen in the presence of Pd(OH)_2_, and the resulting amine was protected with a 2-nitrobenzenesulfonyl (Ns) group to give **5** in 69% yield from **4**. The diamine **5** was linked with 1,2-bis(2-iodoethoxy)ethane in the presence of potassium carbonate to give dimer **6** in 53% yield. After conversion of the two Ns groups into Boc groups by treatment with thiophenol followed by (Boc)_2_O (71% yield in 2 steps), all four Boc groups in **7** were deprotected with TFA to give L2H2-6OTD-dimer (**3**) in 99% yield. With dimer L2H2-6OTD (**3**) in hand, we next examined the interaction mode and efficacy of **3** with the long telomeric DNA models.

*Reagents and conditions*: (**a**) Pd(OH)_2_, H_2_, MeOH: THF (1:1), rt, 5 h; (**b**) NsCl, Et_3_N, CH_2_Cl_2_:MeOH (9:1), −78 °C to rt, 16 h, 2 steps 69%; (**c**) 1,2-bis(2-iodoethoxy)ethane, K_2_CO_3_, DMF, r.t. 23 h, 53%; (**d**) PhSH, K_2_CO_3_, DMF, rt, 1.5 h; then (Boc)_2_O, rt, 12 h, 71%; (**e**) TFA, CH_2_Cl_2_, rt, 3 h, 99%.

**Figure 4:**
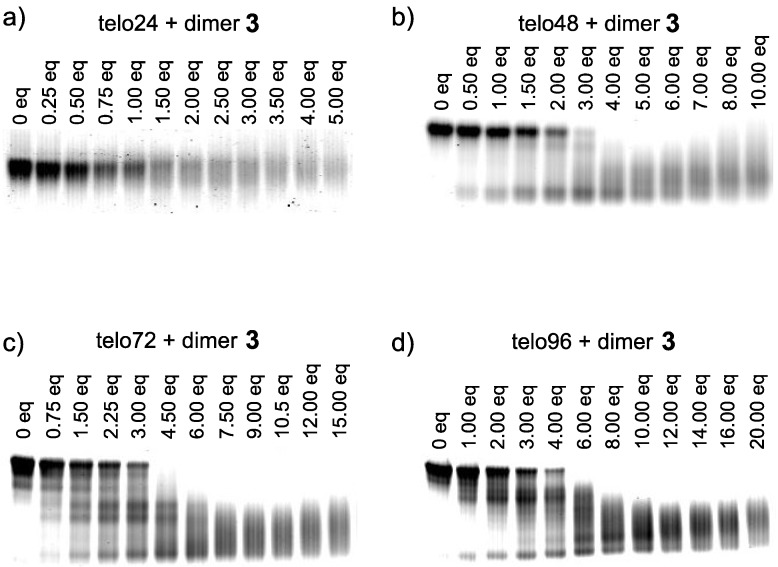
Evaluation of the interaction of telomeric DNAs (**a**) telo24, (**b**) telo48, (**c**) telo72, and (**d**) telo96 with **3 **by EMSA.

First, interaction of L2H2-6OTD-dimer (**3**) with the short telomeric DNA model of telo24 was confirmed by EMSA (Figure 4a). In this case, the original band of telo24 was transformed to a smear with increasing equivalents of dimer **3** [54]. Then, the long telomeric DNA models telo48, telo72 and telo96 were subjected to complexation with dimer **3**, and the results are summarized in Figure 4b–d [55]. In all cases, the original bands of telomeric DNAs migrated further in proportion to the increment of the ligand **3**. Thus, even the longer telomeric DNAs were clearly interacted with dimer type G4 ligand **3**. Since each band converged to a single band with a long migration distance in the presence of a sufficient amount of ligand **3**, the L2H2-6OTD-dimer (**3**) was suggested to form a unimolecular ligand-G4 complex with every size of telomeric DNA examined.

**Figure 5:**
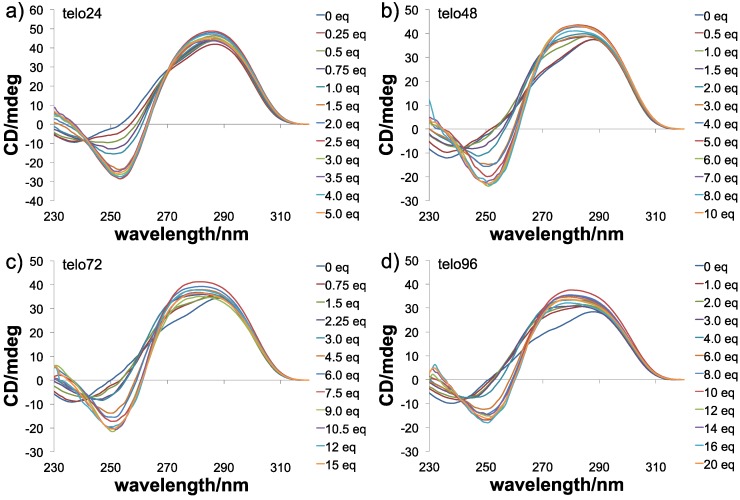
CD spectra of telomeric DNAs in the presence of KCl (100 mM) upon titration with **3**, (**a**) telo24 (10 μM) + **3** (0–5 eq.), (**b**) telo48 (5 μM) + **3** (0–10 eq.), (**c**) telo72 (3.3 μ) + **3** (0–15 eq.) and (**d**) telo96 (2.5 μM) + **3** (0–20 eq.).

Then, the topologies of the G4s obtained from interaction of every size of telomeric DNA with dimer **3** were evaluated by means of CD titration experiments [56]. Telomeric DNAs are known to form multiple G4 topologies, such as parallel, anti-parallel, and hybrid types, and these are usually present as a mixture or in equilibrium, depending upon the conditions [7,8]. When telo24 (10 μM) was titrated with L2H2-6OTD-dimer (**3**), typical spectral changes indicating a change from hybrid-type to anti-parallel topology were observed,* i.e.*, a shoulder at 268 nm disappeared and a positive Cotton effect at 240 nm and a negative effect at 255 nm appeared (Figure 5a). The topologies of the longer DNAs were also examined by CD spectral titration with **3** in the presence of 100 mM KCl. All the longer telomeric DNAs showed spectral changes quite similar to those of telo24, indicating that dimer **3** induced the increment of the anti-parallel form of the longer telomeric DNAs as well (Figure 5). In these experiments, similar degrees of CD intensity (y-axis in Figure 5) were observed with each length of telomeric DNA in the presence of an excess amount of the ligand **3**. Since the concentrations of the DNAs used in the titration were 10 μm for telo24, 5 μm for telo48 (half the concentration of telo24), 3.3 μM for telo72 (one-third of telo24), and 2.5 μm for telo96 (a quarter of telo24), the number of anti-parallel G4 units in telo48, telo72 and telo96 were suggested to be two, three and four, respectively, based upon the case of telo24, which should form one G4 unit (Figure 3).

Next, the stabilization of the topologies obtained from each size of telomeric DNA with 6OTD-dimer **3** was evaluated by means of CD melting experiments, in comparison with the corresponding monomer L2H2-6OTD (**2**). Each size of telomeric DNA,* i.e.*, telo24, telo48, telo72 and telo96, was incubated with **3** (a half equivalent* versus* the monomer **2**), and the melting temperatures (*T*_m_) for those DNAs were determined (Table 1). The *T*_m_ values for telo24, telo48, telo72 and telo96 were found to be 73, 74, 70, and 69 °C, respectively, which are all quite high values compared with that obtained under the ligand-free conditions. In addition, dimer **3** stabilizes every length of telomeric G4 to approximately the same extent as a half equivalent of monomer L2H2-6OTD (**2**) [53].

Finally, we examined the telomerase inhibitory activities of the L2H2-6OTD monomer (**2**) and its dimer **3** in a cell-free system by telomerase repeat amplification protocol (TRAP) assay using PC3 cell lysate [10]. Telomerase is known to elongate various lengths of telomere sequences, and the stabilization ability of each ligand can be evaluated with TRAP assay. In this assay, the monomer **2** and dimer **3** showed telomerase inhibitory activity with IC_50_ values of 15 nM and 7.6 nm, respectively, and the dimer **3** was also found to be more effective than the monomer **2** at the* in vitro* level.

**Table 1:** *T*_m_ values (°C) of telomeric DNAs in the absence or presence of G4 ligands **2** and **3** ^a,b,c^.

DNA oligomers	Ligands	Tm
	absence	59
telo24	**2** (4 eq.)	75
	**3** (2 eq.)	73
	absence	57
telo48	**2** (8 eq.)	76
	**3** (4 eq.)	74
	absence	55
telo48	**2** (12 eq.)	73
	**3** (6 eq.)	70
	absence	55
telo48	**2** (16 eq.)	70
	**3** (8 eq.)	69

^a^* T*_m_ values were obtained by monitoring the CD bands at 295 nm in the presence of KCl (100 mM). ^b^ Melting temperature is defined as the temperature at 0.5 value of normalized CD [mdeg]. ^c^ Values for the monomer **2** were previously reported [53].

## 3. Experimental

### 3.1. General

Flash chromatography was performed on silica gel 60 (spherical, particle size 0.040–0.100 mm; Kanto, Tokyo, Japan). Optical rotations were measured on a P 2200 polarimeter (JASCO, Tokyo, Japan), using the sodium lamp (589 nm). ^1^H and ^13^C-NMR spectra were recorded on JNM-ECX 300 and 400 instruments (JEOL, Tokyo, Japan). The spectra are referenced internally according to the residual solvent signals of CDCl_3_ (^1^H-NMR; *δ* = 7.26 ppm, ^13^C-NMR; *δ* = 77.0 ppm), DMSO-*d*_6_ (^1^H-NMR; *δ* = 2.50 ppm, ^13^C-NMR; *δ* = 39.5 ppm). Data for NMR are recorded as follows; chemical shift (*δ*, ppm), multiplicity (s, singlet; d, doublet; t, triplet; m, multiplet; br, broad), integration, coupling constant (Hz). Data for ^13^C-NMR are reported in terms of chemical shift (*δ*, ppm). Mass spectra were recorded on JEOL JMS-T100X spectrometer with ESI-MS mode using MeOH. 

### 3.2. Synthesis

*Compound* **5**: To a solution of **4** (1.13 g, 1.26 mmol) in MeOH (100 mL) was added Pd(OH)_2_/C (700 mg) and the reaction mixture was stirred at room temperature under an atmosphere of hydrogen gas (balloon). After 5 h, the reaction mixture was filtered through a pad of Celite and filtrates were concentrated *in vacuo* to give the corresponding amine. The crude amine was dissolved in a mixture of CH_2_Cl_2_-MeOH (9:1, 150 mL), and triethylamine (353 μL, 2.53 mmol) and NaCl (560 mg, 2.53 mmol) was added at 78 °C. After being stirred for 16 h, H_2_O was added to the reaction mixture, and the aqueous layer was extracted with CHCl_3_. The extracts were dried over MgSO_4_, filtered, and concentrated *in vacuo*. The residue was purified by silica gel column (EtOAc) to give **5** as a white solid (0.85 g, 0.90 mmol, 69%). [α]^25^_D_ = −18.6 (*c* 1.5, CHCl_3_); ^1^H-NMR (400 MHz, CDCl_3_) *δ* 8.43 (d, *J* = 8.0 Hz, 1H), 8.26 (d, *J* = 8.0 Hz, 1H), 8.23–8.07 (m, 8H), 7.94–7.88 (m, 1H), 7.70–7.63 (m, 1H), 5.46 (t, *J* = 5.5 Hz, 1H), 5.40–5.26 (m, 2H), 4.68 (br, 1H), 3.12–2.88 (m, 4H), 2.08–1.69 (m, 4H), 1.56–0.94 (m, 17H); ^13^C-NMR (100 MHz, CDCl_3_) *δ* 164.6, 164.1, 159.6, 159.5, 155.8, 155.7, 154.6, 154.5, 147.6, 141.0, 140.9, 140.8, 139.4, 139.3, 139.2, 138.7, 138.6, 136.6, 136.4, 134.6, 132.8, 132.7, 130.5, 130.3, 129.3, 129.2, 125.7, 78.7, 47.6, 47.5, 47.4, 47.3, 43.3, 40.1, 34.5, 33.7, 29.3, 28.6, 28.2, 28.1, 21.7, 21.0; HRMS (ESI, M+Na) calcd for C_41_H_41_N_11_O_14_SNa 966.2453, found 966.2413.

*Compound* **6**: To a solution of **5** (1.28 g, 1.36 mmol) in DMF (10 mL) was added K_2_CO_3_ (9.40 g, 68.0 mmol) and 1,2-bis(2-iodoethoxy)ethane (0.25 g, 0.68 mmol) in DMF (1 mL), and the mixture was stirred at room temperature for 23 h. To the reaction mixture was added H_2_O, and aqueous layer was extracted with CHCl_3_. The extracts were dried over MgSO_4_, filtered, and concentrated *in vacuo*. The residue was purified by silica gel column (EtOAc: MeOH = 9: 1) to give **6** (0.75 g, 0.36 mmol, 53%). [α]^25^_D_ = −17.2 (*c* 1.5, CHCl_3_); ^1^H-NMR (300 MHz, CDCl_3_) *δ* 8.61–8.51 (m, 2H), 8.47–8.36 (m, 4H), 8.28–8.05 (m, 16H), 7.88-7.81 (m, 2H), 7.61–7.52 (m, 2H), 5.45–5.25 (m, 4H), 4.63 (br, 2H), 3.59–2.90 (m, 20H), 2.15–1.68 (m, 8H), 1.65–0.62 (m, 34H); ^13^C-NMR (100 MHz, CDCl_3_) *δ* 164.9, 163.9, 159.7, 159.5, 156.0, 155.9, 154.7, 154.6, 147.6, 141.0, 140.9, 139.5, 139.3, 138.5, 136.8, 136.7, 135.6, 132.6, 131.7, 130.9, 130.8, 130.2, 129.6, 129.3, 125.6, 78.9, 77.2, 70.3, 69.7, 47.7, 46.9, 45.0, 40.9, 40.2, 34.8, 33.4, 29.4, 28.3, 26.8, 21.9, 20.2; HRMS (ESI, M+Na) calcd for C_88_H_92_N_22_O_30_S_2_Na 2023.5689, found 2023.5678.

*Compound* **7**: To a solution of **6** (31 mg, 15.4 μmol) in DMF (2 mL) was added K_2_CO_3_ (213 mg, 1,540 μmol) and PhSH (17 μL, 154 μmol), and mixture was stirred at room temperature for 1.5 h. To the reaction mixture was added (Boc)_2_O (68 mg, 308 μmol), and mixture was stirred at room temperature for 12 h. To the reaction mixture was added H_2_O, and aqueous layer was extracted with CHCl_3_. The extracts were dried over MgSO_4_, filtered, and concentrated *in vacuo*. The residue was purified by preparative TLC (CHCl_3_/MeOH = 15: 1) to give **7 **(20 mg, 10.9 μmol, 71%). [α]^25^_D_ = −4.7 (*c* 1.0, CHCl_3_); ^1^H-NMR (400 MHz, CDCl_3_) *δ* 8.59–8.51 (m, 4H), 8.27–8.18 (m, 12H), 5.47–5.32 (m, 4H), 4.63 (br, 2H), 3.85–3.01 (m, 20H), 2.50–1.90 (m, 8H), 1.57–0.79 (m, 52H); ^13^C-NMR (100 MHz, CDCl_3_) *δ* 164.8, 164.7, 159.8, 159.7, 156.0, 155.9, 154.6, 140.9, 139.1, 138.4, 136.8, 130.9, 129.6, 128.8, 79.3, 79.0, 77.2, 70.5, 70.2, 69.6, 69.5, 47.9, 47.8, 40.3, 34.6, 29.7, 29.5, 28.4, 28.3, 21.9; HRMS (ESI, M+Na) calcd for C_88_H_102_N_20_O_26_Na 1853.7172, found 1853.7160.

*L2H2-6OTD-dimer* **3**: To a solution of **7** (18 mg, 9.67 μmol) in CH_2_Cl_2_/TFA (1: 1, 10 mL) was stirred at room temperature for 3 h. The resulting mixture was concentrated *in vacuo* to give **3** (18 mg, 9.56 μmol, 99%). [α]^25^_D_ = +72.0 (*c* 0.6, MeOH); ^1^H-NMR (400 MHz, DMSO-d_6_) *δ* 9.18–9.11 (m, 8H), 8.95–8.91 (m, 4H), 8.60 (br, 2H), 8.35 (d, *J* = 7.3 Hz, 4H), 7.72 (br, 4H), 5.47–5.38 (m, 4H), 3.63–3.45 (m, 8H), 3.09–2.99 (m, 4H), 2.94–2.83 (m, 4H), 2.77–2.69 (m, 4H), 2.14–1.87 (m, 8H), 1.68–0.71 (m, 16H); ^13^C-NMR (100 MHz, DMSO-d_6_) *δ* 164.4, 158.8, 158.7, 155.6, 155.5, 154.5, 154.4, 142.5, 141.9, 141.2, 136.0, 129.7, 128.4, 69.4, 65.5, 47.3, 46.6, 46.0, 38.6, 33.5, 33.3, 26.6, 25.0, 21.2, 20.9; HRMS (ESI, M+Na) calcd for C_66_H_70_N_20_O_18_Na 1453.5075, found 1453.5071.

### 3.3. Electrophoresis Mobility Shift Assay (EMSA)

EMSA was performed as follows; oligonucleotides (2.0 μL, 50 μM of telo24, 25 μM of telo48, 16.7 μM of telo72 and 12.5 μM of telo96) in TE buffer was added to Tris-HCl buffer (5.0 μL, 50 mM, pH 7.1) with 250 mM of KCl. This solution was heated at 94 °C for 2 min and then slowly cooled to 25 °C. Corresponding concentration of **2** and **3** in DMSO was added to the DNA solution. After a 14 h incubation period, the samples were mixed with Ficoll 400 solution (2.0 μL, 100 mg/mL). Next, each mixture (0.5 μL) was run on 12% nondenatured polyacrylamide gels in 1 × TBE buffer (100 V for 10 min, then 200 V for 15 min for telo24, 30 min for telo48, 45 min for telo72 and 60 min for telo96, respectively), and stained with GelStar^®^. The gels were scanned with a phosphorimager (Typhoon 8600, GE Healthcare, Buckinghamshire, UK) using a 580–640 band pass filter.

### 3.4. CD Spectrometry

CD spectra was recorded on a J-720 spectropolarimeter (JASCO, Tokyo, Japan) using a quartz cell of 1 mm optical path length and an instrument scanning speed of 500 nm/min with a response time of 1 s, over a wavelength range of 220–320 nm. The nucleotides purified (Sigma Genosys, Hokkaido, Japan) were dissolved as 1.0 mM stock solutions in sterilized water to be used without further purification. These nucleotides were diluted to 10 μM (telo24), 5.0 μM (telo48), 3.3 μM (telo72) and 2.5 μM (telo96) with 50 mM Tris–HCl, pH 7.5, 100 mM KCl buffer, respectively. Subsequently, these solution were annealed by heating at 96 °C for 2 min, then slowly cooled to room temperature, and then corresponding concentrations of **2** and **3** were titrated into these DNA solutions. Finally the CD spectra are representative of ten scans taken at 25 °C.

### 3.5. CD Melting Experiments

A solution of all DNA (10 *μ*M: telo24, 5.0 *μ*M: telo48, 3.3 *μ*M: telo72, 2.5 *μ*M: telo96) was prepared in 50 mM Tris–HCl, pH 7.5, 100 mM KCl buffer. Subsequently, corresponding concentration of **2** and** 3** were added. Melting curves were obtained by monitoring the CD intensity at 295 nm on a JASCO J-720 spectropolarimeter using a quartz cell of 1 mm optical path length; the temperature was changed as follows: 25 to 99 °C then 99 to 25 °C at 1.0 °C/min.

### 3.6. Telomerase Repeat Amplification Protocol (TRAP) Assay

Telomerase repeat amplification protocol (TRAP) assays were conducted as follows; 1 × 10^5^ of PC3 cells were lysed with 200 μL of TRAP lysis buffer [57], placed on ice for 30 min, and centrifuged for 20 min (15,000 rpm, 4 °C). This TRAP lysate (2.0 μL) was mixed with 10×T-PCR buffer (5.0 μL), 2.5 mM dNTPs (1.0 μL), 50 ng/μL TS primer (5'-d[AATCCGTCGAGCAGAGTT]-3', 2.0 μL), 0.2 pM TSNT (5'-d[AATCCGTCGAGCGAGTTAAAAGGCCGAGAAGCGAT]-3', 0.5 μL), DEPC (Diethylpyro carbonate) solution in MilliQ water (30 μL), compounds (DMSO solution, 5.0 μL), and placed on ice for 30 min. After the telomerase reactions (30 min, 20 °C), the reacting solution was placed on ice, 2.0 μL of 10 μM NT primer (5'-d[ATCGCTTCTCGGCCTTTT]-3’), 2.0 μL of 10 μL ACX primer (5'-d[GCGCGG (CTTACC)_3_CTAACC]-3') and 0.5 μL of Gene Taq (Nippon Gene, Tokyo, Japan) were added. The three-step PCR was performed: 27 cycles of 94 °C for 25 s, 50 °C for 25 s, and 72 °C for 45 s. PC3 cell lysates were used as a negative control, and lysis buffer as a positive control. Approximately 5.0 μL of each PCR reacting solution and 1.0 μL of 10 × loading buffer (Takara Bio Inc., Shiga, Japan) were loaded onto a 10% nondenaturing polyacrylamide gel (Perfect NT Gel, D.R.C. Co., Ltd., Tokyo, Japan) and run for 10 min at 100 V, then 55 min at 200 V. Gels were stained with SYBR^®^ Green I (Takara Bio Inc., Shiga, Japan), and visualized with Fuji FLA-2000 (Fujifilm, Tokyo, Japan). Each sample was examined in duplicate by the TRAP assay on different occasions and the reproducibility was confirmed. IC_50_ value was calculated from average of two independent experiments.

## 4. Conclusions

In summary, we have newly synthesized a dimer-type G4 ligand **3**, and evaluated its binding properties with long telomeric DNAs, telo48, 72 and 96, by means of EMSA and CD titration. The L2H2-6OTD-dimer (**3**) interacted with the long telomeric DNAs by forming an anti-parallel type G4 structure with each 24-base unit. The stabilization efficacy of **3** with long telomeric DNAs was superior to that of the monomer L2H2-6OTD (**2**) at both the molecular level (CD melting assay) and the enzymatic level (TRAP assay). 

## Supplementary Materials

Supplementary materials can be accessed at: http://www.mdpi.com/1420-3049/18/4/4328/s1.
