# Supplementary material for: Evaluation of the Interaction between Long Telomeric DNA and Macrocyclic Hexaoxazole (6OTD) Dimer of a G-quadruplex Ligand
Source: Molecules. 2013 Apr 12;18(4):4328–41. doi: 10.3390/molecules18044328 (PMC6269967; doi:10.3390/molecules18044328)
Supplement: Supplementary file 1 [file molecules-18-04328-s001.pdf]

Supplementary Materials

Figure S1. Copies of NMR spectra of compounds (**3**–**7**).

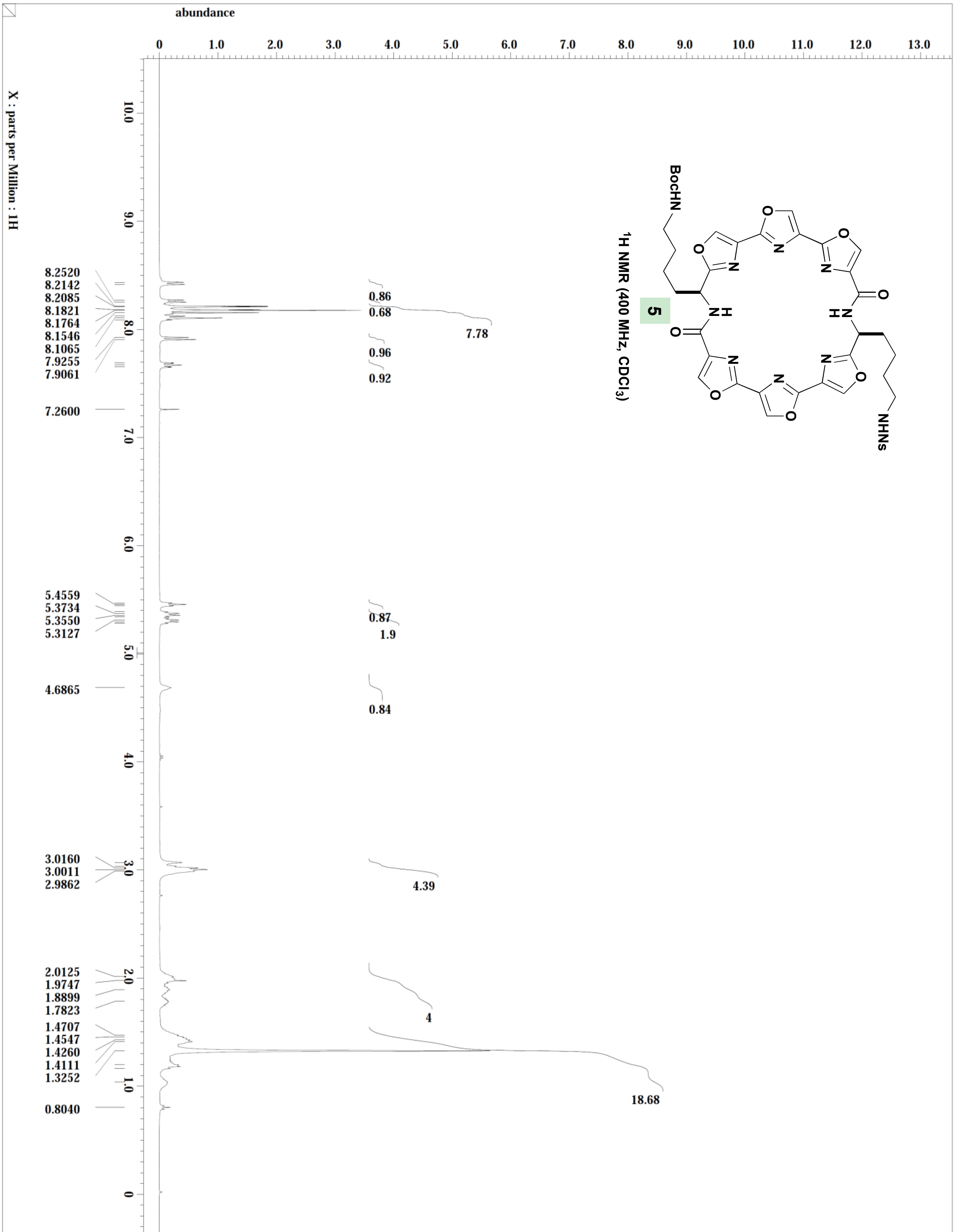

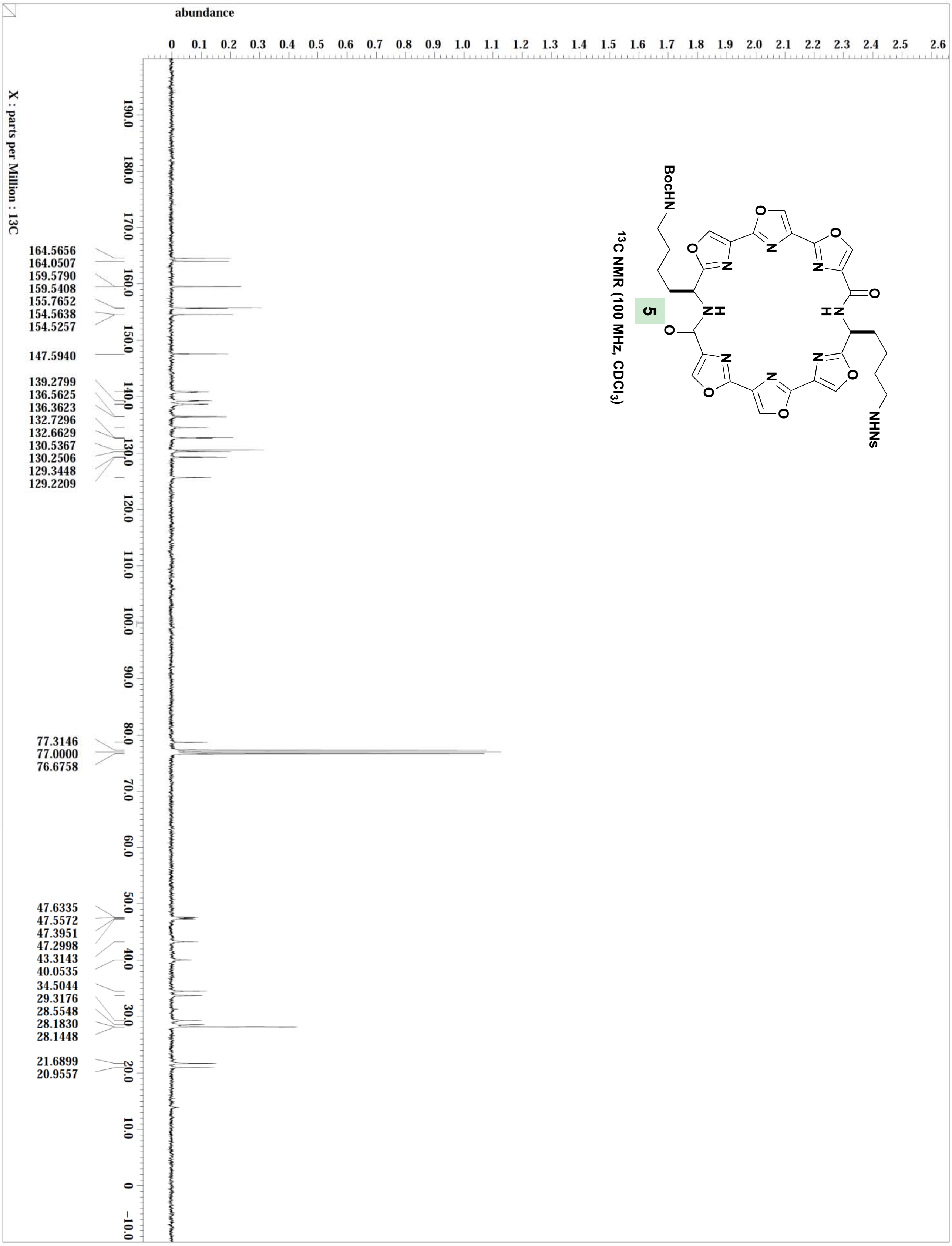

abundance
X : parts per Million : 13C
164.5656
164.0507
159.5790
159.5408
155.7652
154.5638
154.5257
147.5940
139.2799
136.5625
136.3623
132.7296
132.6629
130.5367
130.2506
129.3448
129.2209
77.3146
77.0000
76.6758
47.6335
47.5572
47.3951
47.2998
43.3143
40.0535
34.5044
29.3176
28.5548
28.1830
28.1448
21.6899
20.9557
BocHN
NHNs
5
13C NMR (100 MHz, CDCl3)

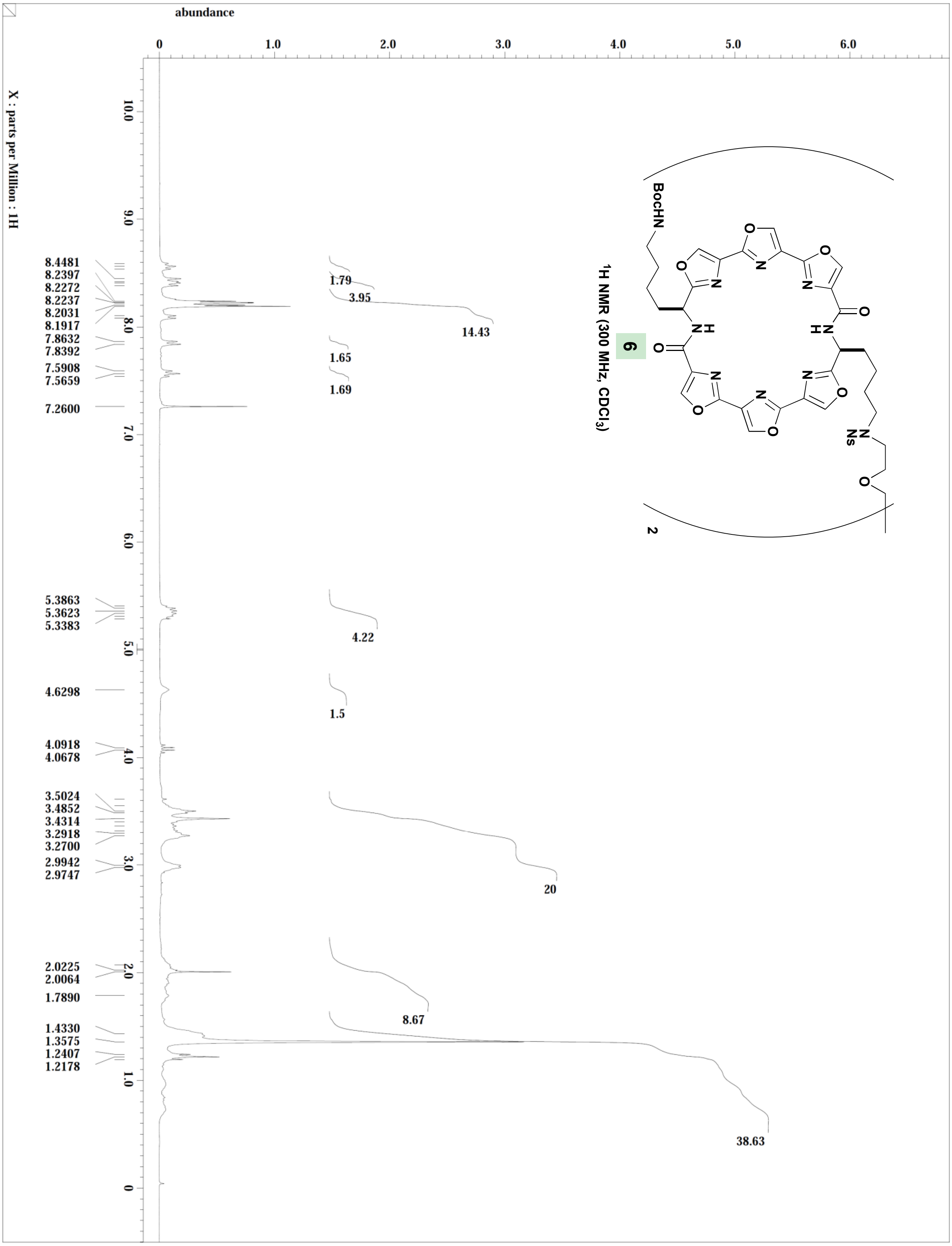
abundance
X : parts per Million : 1H
1H NMR (300 MHz, CDCl3)
6
BocHN
Ns
2
8.4481
8.2397
8.2272
8.2237
8.2031
8.1917
7.8632
7.8392
7.5908
7.5659
7.2600
5.3863
5.3623
5.3383
4.6298
4.0918
4.0678
3.5024
3.4852
3.4314
3.2918
3.2700
2.9942
2.9747
2.0225
2.0064
1.7890
1.4330
1.3575
1.2407
1.2178
1.79
3.95
14.43
1.65
1.69
4.22
1.5
20
8.67
38.63

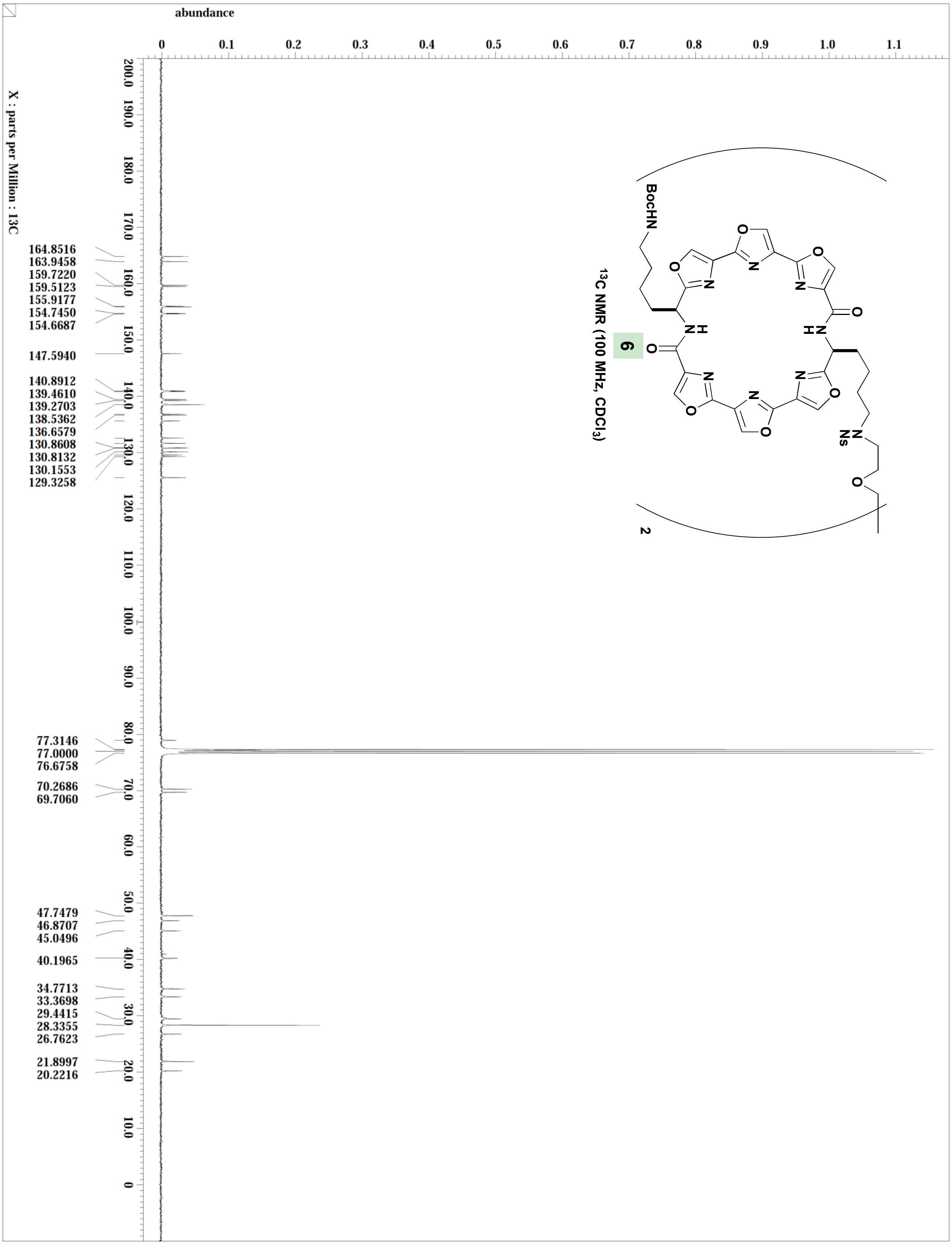
abundance
X : parts per Million : 13C
164.8516
163.9458
159.7220
159.5123
155.9177
154.7450
154.6687
147.5940
140.8912
139.4610
139.2703
138.5362
136.6579
130.8608
130.8132
130.1553
129.3258
77.3146
77.0000
76.6758
70.2686
69.7060
47.7479
46.8707
45.0496
40.1965
34.7713
33.3698
29.4415
28.3355
26.7623
21.8997
20.2216
BocHN
Ns
6
2
13C NMR (100 MHz, CDCl3)

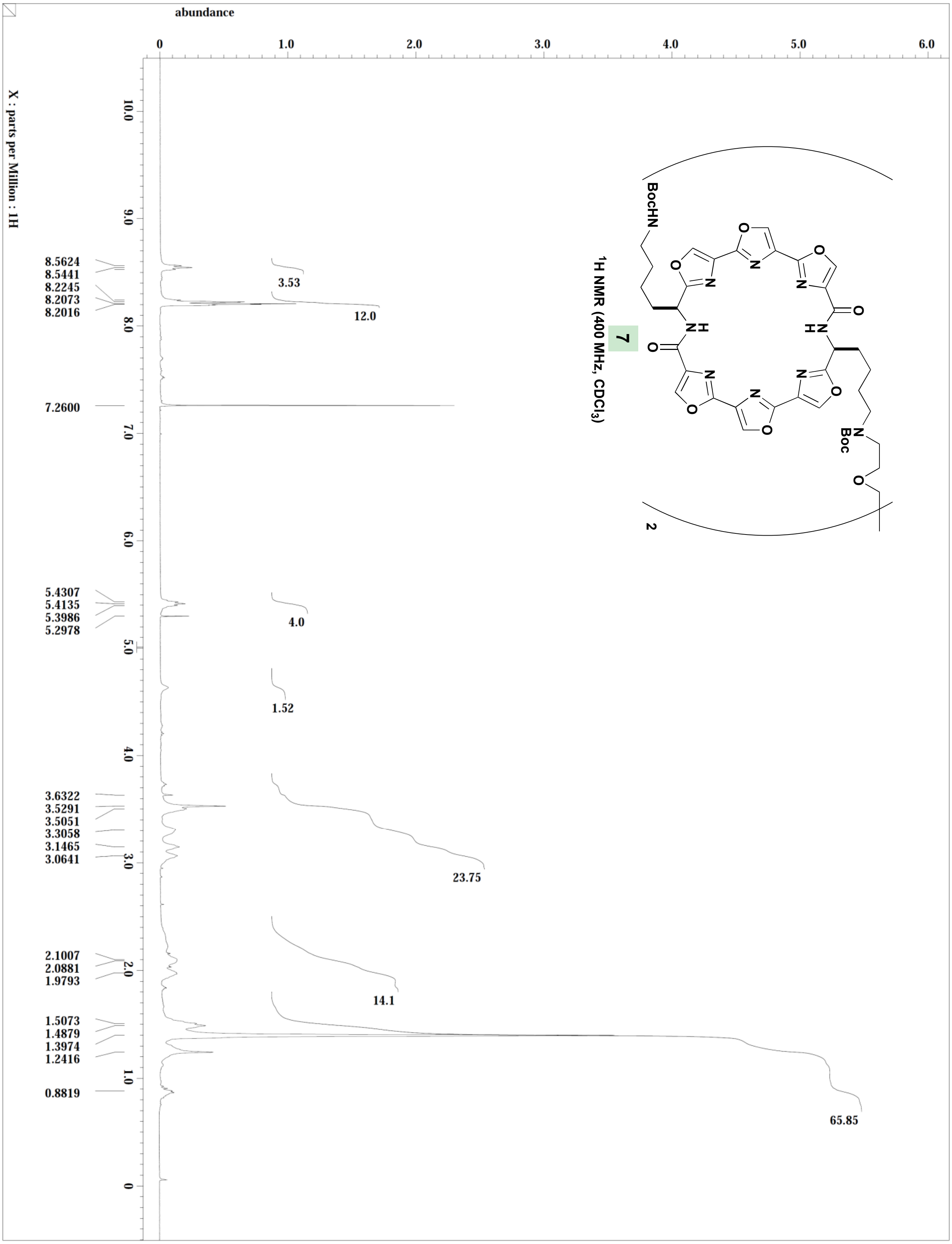
abundance
0
1.0
2.0
3.0
4.0
5.0
6.0
X : parts per Million : 1H
10.0
9.0
8.0
7.0
6.0
5.0
4.0
3.0
2.0
1.0
0
8.5624
8.5441
8.2245
8.2073
8.2016
7.2600
5.4307
5.4135
5.3986
5.2978
3.6322
3.5291
3.5051
3.3058
3.1465
3.0641
2.1007
2.0881
1.9793
1.5073
1.4879
1.3974
1.2416
0.8819
3.53
12.0
4.0
1.52
23.75
14.1
65.85
1H NMR (400 MHz, CDCl3)
7
BocHN
Boc
2

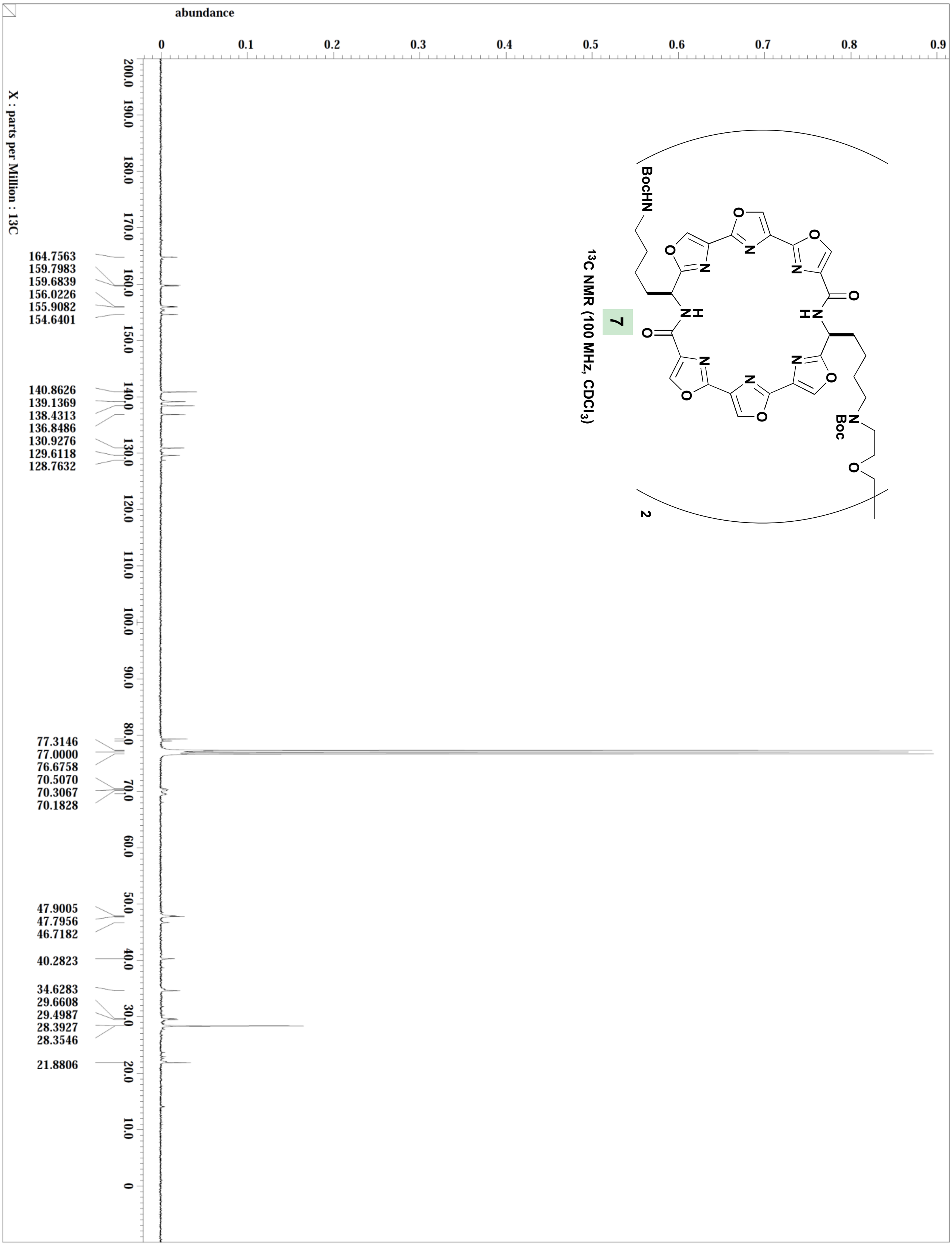

abundance
X : parts per Million : 13C
164.7563
159.7983
159.6839
156.0226
155.9082
154.6401
140.8626
139.1369
138.4313
136.8486
130.9276
129.6118
128.7632
77.3146
77.0000
76.6758
70.5070
70.3067
70.1828
47.9005
47.7956
46.7182
40.2823
34.6283
29.6608
29.4987
28.3927
28.3546
21.8806
7
13C NMR (100 MHz, CDCl3)
BocHN
Boc

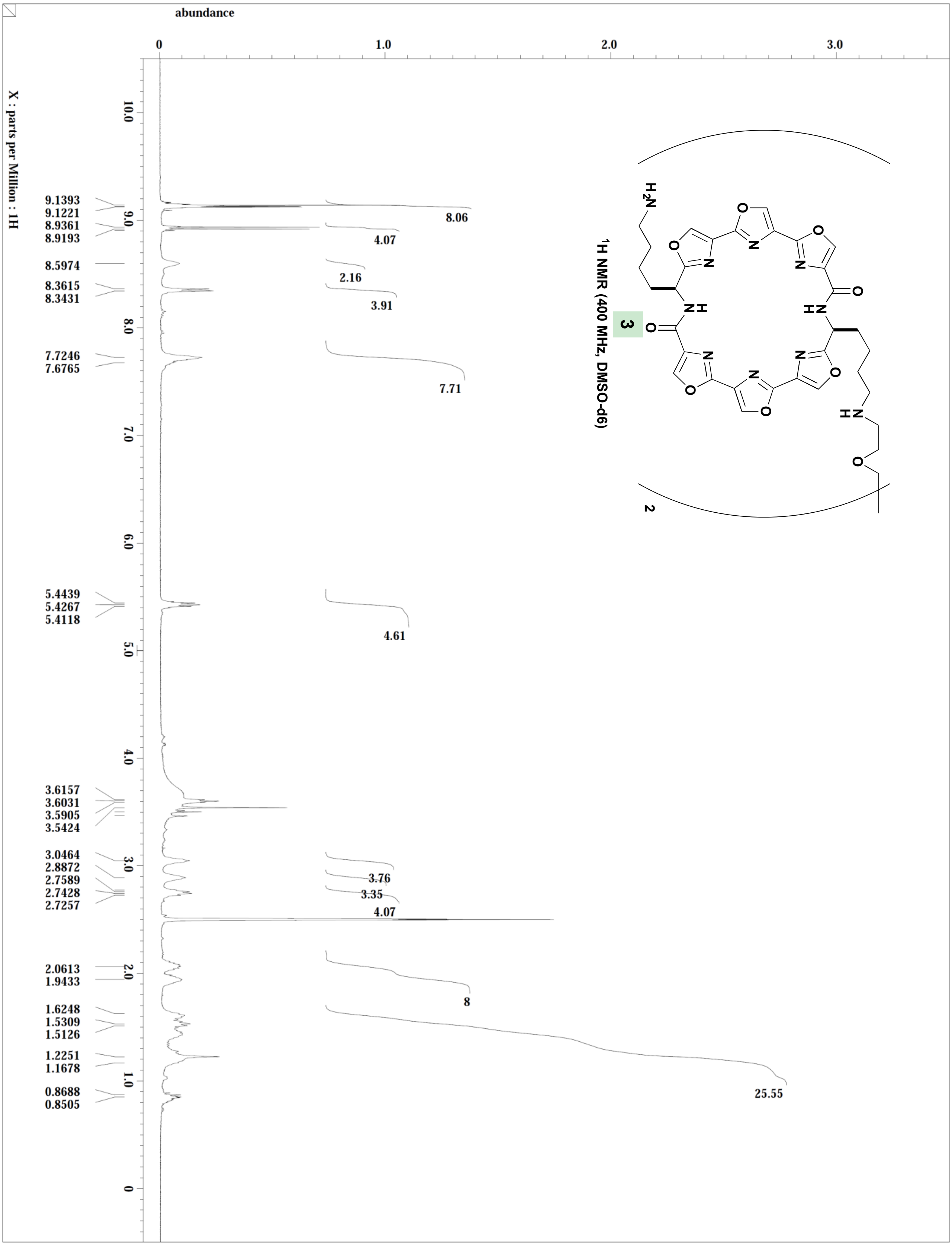
abundance
X : parts per Million : 1H
^{1}H NMR (400 MHz, DMSO-d6)
3

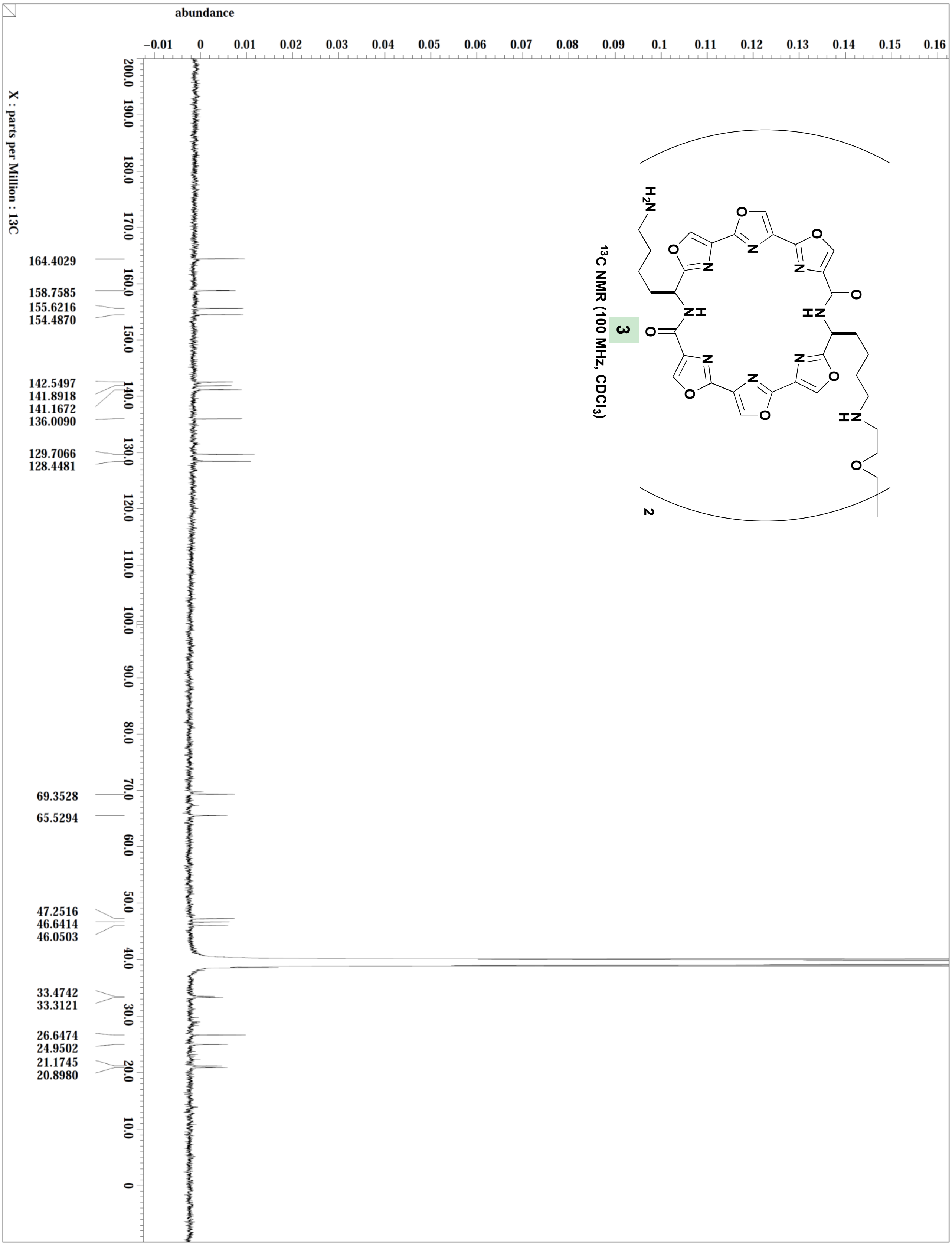
abundance
X : parts per Million : 13C
3
13C NMR (100 MHz, CDCl3)
164.4029
158.7585
155.6216
154.4870
142.5497
141.8918
141.1672
136.0090
129.7066
128.4481
69.3528
65.5294
47.2516
46.6414
46.0503
33.4742
33.3121
26.6474
24.9502
21.1745
20.8980

ESI-MS Spectrometry

All measurements were carried out on a JMS-T100LC AccuTOF (JEOL), using the electrospray ionization (ESI) source in negative mode, as described previously. The measurement conditions and the sample preparation procedures were as follows: capillary needle voltage, −2.0 kV; ring lens voltage, −15 V; orifice 1 voltage, −75 V; orifice 2 voltage, 0 V; orifice 1 temperature, 80 °C; desolvation temperature, 80 °C; sample flow rate, 5 mL min^{-1}; All experiments were performed in 20 mM NH_4OAc containing 10 µM of GFOs and 40 µM of **7**. Methanol (10%) was added just before injection. The role of methanol is to increase ion signals.

Figure S2. ESI mass spectra of 10 µM telo24 with 40 µM dimer **3**.

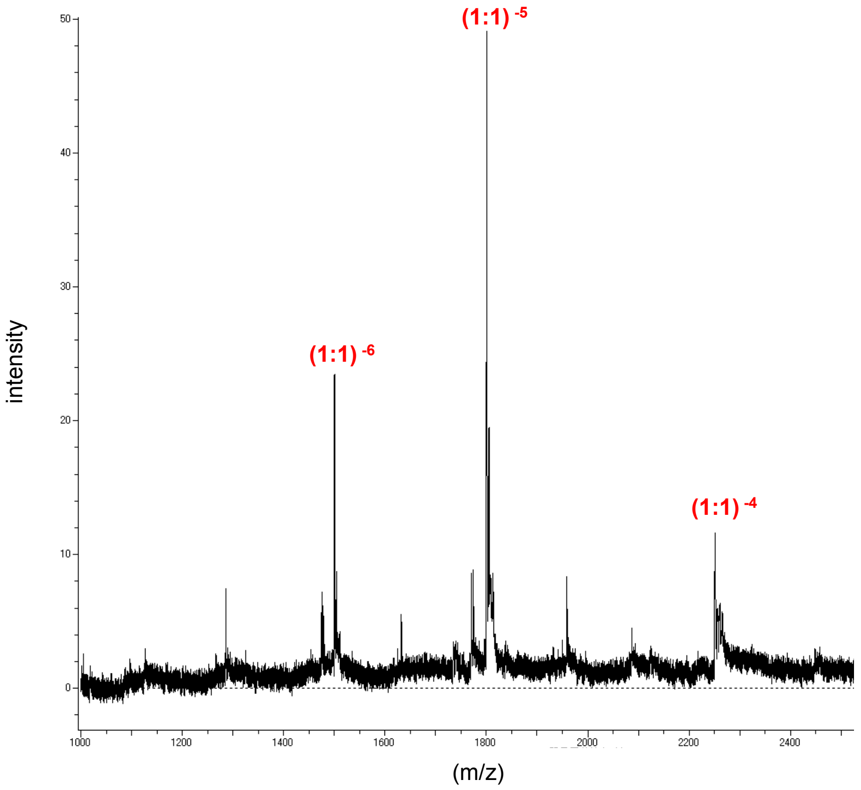

Telomeric DNA $(TTAGGG)_4$	10 µM
Dimer (**7**)	40 µM
NH_4OAc	20 mM

(one DNA + one dimer-4H+)$^{-4}$ = calct. 2250.44, found 2251.12
(one DNA + one dimer-5H+)$^{-5}$ = calct. 1799.95, found 1800.76
(one DNA + one dimer-6H+)$^{-6}$ = calct. 1499.79, found 1500.47

Figure S3. Normalized thermal melting and annealing profiles recorded at 295 nm of (TTAGGG) n (n = 4–16) in the presence of 100 mM KCl with **2**.

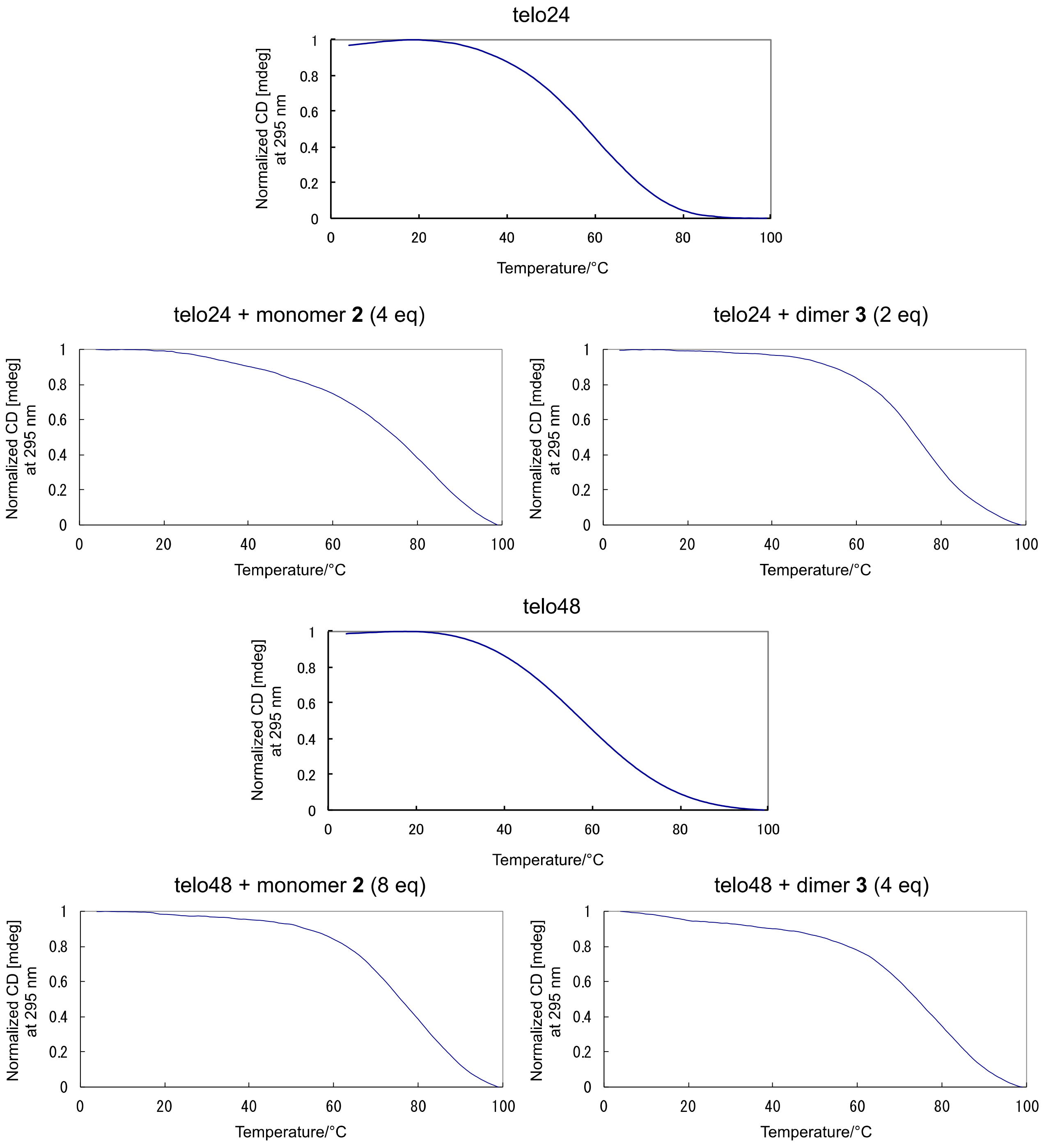

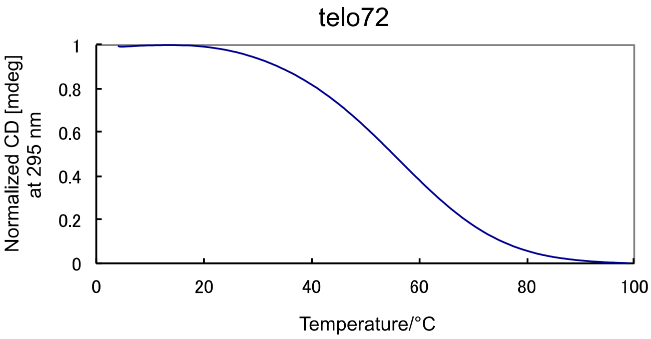
telo72
Normalized CD [mdeg] at 295 nm
Temperature/°C

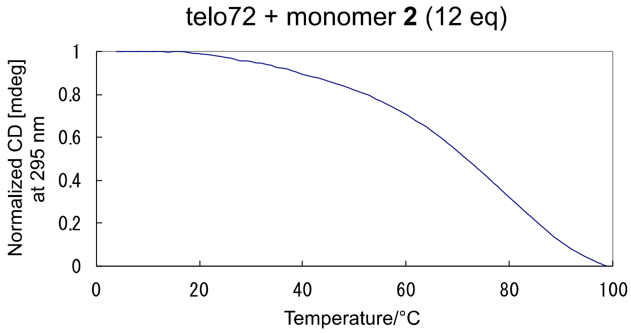
telo72 + monomer **2** (12 eq)
Normalized CD [mdeg] at 295 nm
Temperature/°C

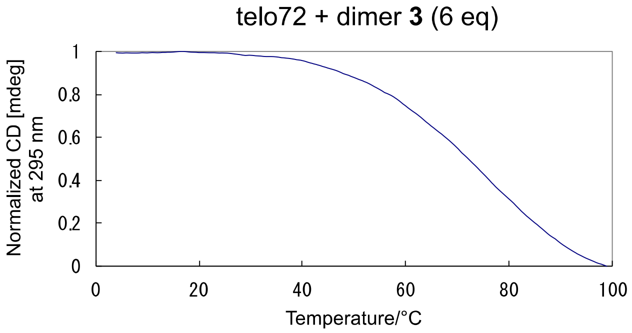
telo72 + dimer **3** (6 eq)
Normalized CD [mdeg] at 295 nm
Temperature/°C

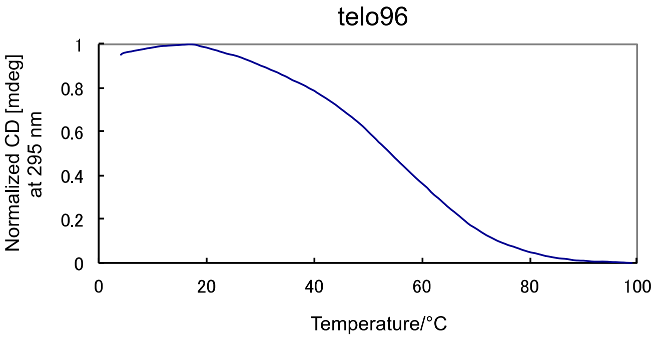
telo96
Normalized CD [mdeg] at 295 nm
Temperature/°C

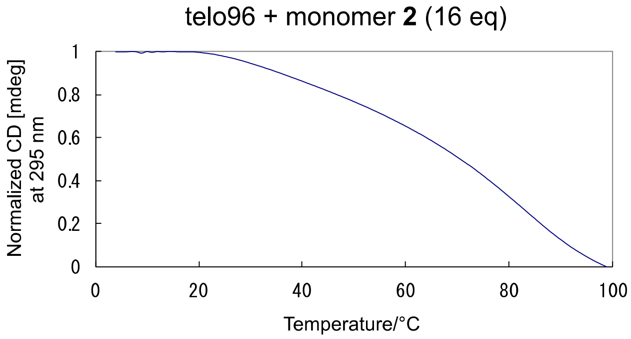
telo96 + monomer **2** (16 eq)
Normalized CD [mdeg] at 295 nm
Temperature/°C

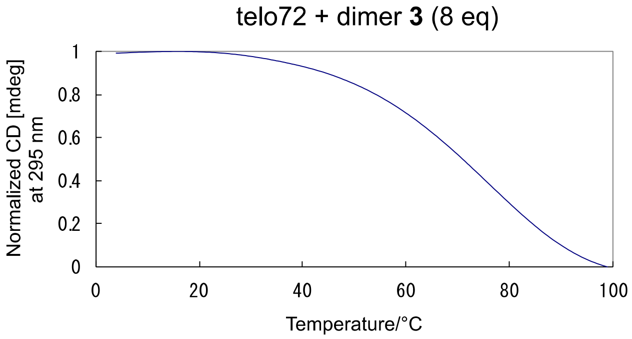
telo72 + dimer **3** (8 eq)
Normalized CD [mdeg] at 295 nm
Temperature/°C

Telomerase Repeat Amplification Protocol (TRAP) Assay

Figure S4. Telomerase inhibitory activity of dimer **3** using TRAP assay.

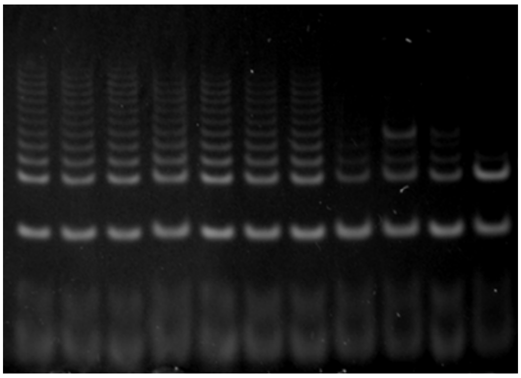

entry 1: No ligands; entry 2, 3 (0.3 nM); entry 4, 5 (1.0 nM); entry 6, 7 (3.0 nM); entry 8, 9 (10 nM); entry 10, 11 (30 nM)
